# Assessing the Impact of Once-Weekly Semaglutide on Glycemic Control in Psychiatric Outpatients with Type 2 Diabetes: A 4-Year Observational Study

**DOI:** 10.1192/j.eurpsy.2026.10453

**Published:** 2026-07-31

**Authors:** P. Argitis, F. E. Kakavitsas, M. Demetriou, M. Peyioti, Z. Chaviaras

**Affiliations:** 1 Psychiatric, General Hospital of Corfu; 2 General practitioner, Health center of Corfu, Corfu, Greece; 3 Psychiatric, General Hospital of Corfu, Corfu, Cyprus; 4 Psychiatric, University Hospital of Alexandroupoli, Corfu, Greece

## Abstract

**Introduction:**

Patients with severe mental illness (SMI), particularly schizophrenia and schizoaffective disorder, experience disproportionately high rates of metabolic syndrome. This stems from lifestyle factors such as sedentary behavior, poor diet, and tobacco use, compounded by reduced access to medical care. Adding to this vulnerability, second-generation antipsychotics—though essential for symptom control—commonly cause weight gain, insulin resistance, and type 2 diabetes mellitus (T2DM). Together, these factors contribute to markedly higher cardiometabolic risk and a life expectancy shortened by over a decade.

**Objectives:**

A particularly challenging aspect of managing T2DM in psychiatric populations is low adherence to complex daily medication regimens. Cognitive deficits, impaired executive functioning, and motivational barriers, all common in psychotic disorders, further hinder consistent self-management. Simplifying treatment through long-acting medications may offer a pragmatic solution. This prospective observational study investigated whether switching from daily oral hypoglycemics to once-weekly subcutaneous semaglutide could improve metabolic outcomes in adults with psychotic disorders and T2DM.

**Methods:**

From January 2021 to January 2025, 87 psychiatric outpatients with comorbid schizophrenia or schizoaffective disorder and T2DM were recruited from a community mental health service. Eligible participants had HbA1c values between 6.5% and 7.4% despite consistent oral antidiabetic therapy and had been on stable antipsychotic treatment for at least three months. All participants were transitioned to once-weekly semaglutide monotherapy, with no changes to psychiatric medication during the 12-month follow-up. HbA1c levels were measured at baseline and again at 3, 6, 9, and 12 months. Patients were instructed to maintain their usual lifestyle and dietary patterns throughout the study period.

**Results:**

Significant reductions in HbA1c were observed. The Friedman test showed a time effect (χ²(3) = 18.74, p < 0.001), with Wilcoxon tests confirming reductions from baseline to 6 months (Z = –5.24, p < 0.001) and from baseline to 12 months (Z = –4.57, p < 0.001). While 52 patients (59.8%) achieved a ≥0.5% HbA1c reduction by month 6, and 15 (17.2%) exceeded 1.0% reduction by month 12, a small subset (12.6%) experienced worsening glycemic control between months 9 and 12, likely reflecting prior good adherence to oral therapy.

**Conclusions:**

This study shows that once-weekly semaglutide is a practical option for managing T2DM in people with SMI, improving glycemic control without affecting psychiatric stability. For psychiatrists, it highlights the value of integrated somatic care and simplified regimens that overcome self-management barriers in psychosis, stressing the need for cross-disciplinary collaboration in long-term care.

**Disclosure of Interest:**

None Declared

